# Outcomes of miniaturized percutaneous nephrolitotomy in infants: single centre experience

**DOI:** 10.1590/S1677-5538.IBJU.2016.0629

**Published:** 2017

**Authors:** Eyyup Sabri Pelit, Bülent Kati, Cengiz Çanakci, Süleyman Sağir, Halil Çiftçi

**Affiliations:** 1 Department of Urology, Harran University Faculty of Medicine, Sanliurfa, Turkey; 2 Bolvadin State Hospital, Istanbul, Turkey

**Keywords:** Nephrostomy, Percutaneous, Kidney Calculi, Infant

## Abstract

**Objectives:**

The present study was aim to evaluate the safety and efficacy of Mini-PNL to treat kidney stones in patients aged <3 years. This is the one of the largest series in the literature in this age group of patients.

**Material and methods:**

From May 2012 to April 2016, the medical records of 74 infant patients who underwent mini-PNL for renal stones were reviewed retrospectively. All infants were evaluated with the plain abdominal radiograph, urinary ultrasound, non-contrast computerized tomography and/or intravenous urogram. Pre-operative, intraoperative and post-operative data were analyzed.

**Results:**

A total of 74 infant (42 male, 32 female) with a mean age 21.5±8.2 (10-36) months were included in this study. The mean size of the stones was 22.0±5.9 (14-45) mm. A 17 Fr rigid pediatric nephroscope with a pneumatic intracorporeal lithotripsy were used through 20-22 Fr access sheath. The stone-free rate was 84.7% at 1 month after the operation. Mean operative time was 74.0 (40-140) min. Mean fluoroscopy screening time was as 4.3(3.1-8.6) min. Average hospitalization time was 3.8 (2-9) day. Auxiliary procedures were performed to 11(15.3%) patients (7 extracorporeal shock wave lithotripsy, 3 re- percutaneous nephrolitotomy, 1 retrograde intrarenal surgery). No major complication classified as Clavien IV-V observed in study group.

**Conclusions:**

Mini-PNL with pneumatic intracorporeal lithotripsy can be performed safely and effectively to manage kidney stones in infants with high stone free rate and low complications.

## INTRODUCTION

Childhood urolithiasis is a major health problem in developing countries especially in endemic regions. The incidence of childhood urolithiasis ranges between 4-6% and the incidence increases up to 14.8% in endemic regions of the World such as Turkey (1, 2). Pediatric patients generally have underlying metabolic, anatomical, functional abnormalities and/or recurrent urinary tract infections that cause urinary stone. Children with kidney stone are classified as high-risk patients for recurrence and requirement of multiple interventions (3).

In this century, with the advancement of technology, minimal invasive treatment modalities such as extracorporeal shock wave therapy (ESWL), percutaneous nephrolitotomy (PCNL) (micro-, mini-), flexible ureteroscopy (f-URS) are routinely used for the treatment of pediatric renal stones (4). Open surgery is still an option for stones with anatomic renal abnormalities (5). According to the European Urology Guidelines (EAU) for the treatment of pediatric kidney stones, ESWL is the first treatment option for stones smaller than 2cm. Although it is the least invasive method it may require more auxiliary procedures than the other methods and most of the pediatric patients requires anesthesia during the ESWL procedure (6). PCNL should be the preferred treatment method in stones larger than 2cm and ESWL resistant hard stones (7). Since the first series of mini-PCNL technique was published in 1998, mini-PCNL has reached higher stone free rates (SFR) in pediatric patients with lower complication rates (8).

In published literature, there are few studies about mini-PCNL in infancy and number of patients included in these studies are limited. The present study aims to evaluate the safety and efficacy of mini-PNL to treat kidney stones in patients aged <3 years. To our best knowledge, this study is one of the largest series in the literature in this age group of patients.

## MATERIALS AND METHODS

From May 2012 to April 2016, the medical records of 72 infant (42 boys, 30 girls) patients who underwent mini-PNL for renal stones were reviewed retrospectively in a referral tertiary institution in Turkey. The presence of renal stones larger than 2cm, history of previous unsuccessful ESWL and ESWL resistant stones smaller than 2cm were accepted as indications for mini-PNL. ESWL resistance was accepted as two ESWL session failures. Patients who have stones with renal congenital anomaly such as ureteropelvic junction obstruction were excluded from the study. Serum biochemistry, complete blood count, urine analysis and urine culture were performed for all patients prior to surgery. All infants were evaluated with plain abdominal radiography (KUB), urinary ultrasound (USG), non-contrast computerized tomography (NCCT) and/or intravenous urogram (IVP). All patients had sterile urine culture prior to surgery. Urinary tract infection was treated according to bio-sensitivity result of the urine culture. Stone size was accepted as the longest axis measured on NCCT and if multiple stones exists, stone burden was assessed as the sum of longest diameter of each stone.

All patients received intravenous antibiotic prophylaxis 1 hour before the surgery. Patients were placed in the low lithotomy position on heater blanket laid operation Table. Irrigation fluids at room temperature and heater blanket were used to prevent hypothermia in infants. Initially, 3-4Fr open end ureteric catheter was placed into the renal collecting system via semirigid pediatric URS (6Fr Karl Storz, Germany) or pediatric cystoscopy (12Fr Karl Storz, Germany) under fluoroscopic control. Foley urethral catheter (8-10Fr) was used to fix the ureteric catheters. The patient was then turned into the prone position with chest padding not to disturb ventilation and perfusion. Lead aprons were placed over the patient's gonadal region to protect gonads from radiation exposure. Caliceal system of the kidney was punctured with 18g diamond tip needle after opacification of collecting system with retrograde injection of contrast agent under fluoroscopy guidance. A guidewire was inserted to the renal collecting system through the needle and after certification of guidewire location in the renal collecting system, Amplatz renal dilatators were used for tract dilatation up to 20-22Fr. Pneumatic lithotripter 17F nephroscope (Karl Storz, Germany) was used for fragmentation of the stones. Stone fragments were collected with grasping forceps. At the end of the operation renal collecting system was checked for residual stone fragmentation via fluoroscopy; a 10-12Fr nephrostomy catheter was subsequently inserted into the renal pelvis. Antegrade pyelography was performed through the nephrostomy catheter to control the extravasation of the urine. Operation time was clocked from insertion of ureteral catheter to the nephrostomy tube placement. We performed USG and KUB in the 1st and/or 3rd months of follow-up. The frequency of visits and imaging method (BT/USG and KUB) to be used in each visit was determined according to residual fragment burden, localization and presence of obstruction and symptoms of patients during follow-up. Procedure was accepted as stone-free when there was no residual fragmentation on radiological imaging method on 3-month follow-up. Metabolic assessment was performed in all stone free patients at 1 month postoperatively. Prophylaxis was started according to the results of stone analysis and/or metabolic assessment.

All statistical analyses were conducted by using SPSS statistical software (version 15.0; SPSS, Inc., Chicago, IL, USA). A probability value (p value) of <05 was considered statistically significant.

## RESULTS

A total of 72 infants (42 male, 30 female) with a mean age 21.5±8.2 (10-36) months were included in this study. The mean size of the stones was 22.0±5.9 (14-45) mm. Renal stones were located in renal pelvis (n=20), lower pole (n=17), middle pole/upper pole (n=11), all calyx (n=24). Patients had no hydronephrosis (n=12), grade 1(n=15), grade 2 (n=37) and grade 3 hydronephrosis (n=8). All intrarenal access was performed in the prone position and under fluoroscopic guidance. Mean operative time was 69.0 (40-140) min. Mean fluoroscopy screening time was 4.3 (3.1-8.6) min. The stone-free rate was 84.7% at 1 month after the operation. Nephrostomy tube was not inserted postoperatively in 6 (8.4%) patients with no residual stone, extravasation and perioperative hemorrhage and especially in single access, short-running procedures. Auxiliary procedures were performed to 11(15.3%) patients (7 ESWL, 3 re- PCNL, 1 RIRS). Seven out of these 11 patients were completely stone free and these additional procedures increased the overall success rate from 84.7% to 94.4%. The stone size was 15±4.2mm in the clinically successful procedures and 22±4.9 in the failed procedures (P=0.004). Additionally, renal pelvis or single calyceal location was 73.7% in the clinically successful procedures, whereas it was 27.2% in the failed procedures (P=0.002). Four patients were followed via ultrasonography for insignificant fragments. Average hospitalization time was 3.0 (2-9) days. Complications classified as Clavien IV-V were not observed, however one major complication classified as Clavien III was observed in the study group. Five patients required blood transfusions. Extravasation of urine to the retroperitoneum then pleura after withdrawal of the nephrostomy tube was observed in 1 infant and in this patient spontaneous resolution was observed after DJ stent insertion. Bowel perforation was seen in 1 patient which was diagnosed when colonic content was seen in nephrostomy tube and perforated area of descending colon was primarily repaired on postoperative day 3; 1 patient had hydrothorax due to pleural injury during the upper pole access and thorax tube was inserted. Seven patients developed urinary infections and they were treated according to antibiogram results of the urinary culture.

Nine uric acid stones, 11 cystine stones, 16 calcium oxalate-calcium phosphate stones and 7 struvite stones were detected during the postoperative stone analysis Stone composition was not known in 29 patients because of the discontinuation of the follow-up. Demographics, preoperative, intraoperative, postoperative findings of patients, stone composition and factors that affect the stone free status of the patients are summarized in Tables 1-4.

**Table 1:** Patients’ demographics and preoperative data.

Age of patients (months)		21.5±8.2 (10-36)
	Male/Female	42/30 (58.3% / 41.7%)
**Stone size (mm)**		22±5.9 (14-45)
	<20 mm	49 (68.1%)
	>20 mm	23 (31.9%)
**Stone location**		
	Renal pelvis	20 (27.8%)
	Lower pole	17 (23.6%)
	Middle pole/Upper pole	11 (15.3%)
	Partial/complete	24 (33.3%)
staghorn		
	Laterality L/R	41/31
**Hydronephrosis**		
	Grade 0	12 (16.6%)
	Grade 1	15 (20.8%)
	Grade 2	37 (51.4%)
	Grade 3	8 (11.2%)

**Table 2:** Intraoperative data.

Puncture location		
	Subcostal	67(93.1%)
	Supracostal	5 (6.9%)
**Number of puncture**		
	Single	68 (94.4%)
	Multiple	4 (5.6%)
Operative time (mean mins)		69 (40-140)
Hospitalization time (mean days)		3 (2-9)
Fluoroscopic screening time (mean mins)		3.6(1.2-9.8)
Tubeless PNL		6 (8.4%)
Tube PNL		66(91.6%)

**Table 3:** Postoperative data.

Initial Stone free rate(after 1 month)		61 (84.7%)
Stone free rate after additional therapy		68(94.4%)
**Additional procedures**		
	ESWL	7
	Re-PNL	3
	URS	1
Minor (Clavien I-II) complications		14 (19.4%)
Major (Clavien III-V) complications		1 (1.3%)
Preoperative hemoglobin level		12.3 (9.8-15.3)
Postoperative hemoglobin level		11.3 (8.9-13.9)
**Stone composition**		
	Uric acid	9 (12.5%)
	Cystine	11 (15.3%)
	CaOx-CaP	16 (22.3%)
	Struvite	7 (9.7%)
	Unknown	29 (40.2%)

**Table 4:** Factors that affect the stone free status of the patients.

		Initional stone-free status (84.7%)	Residual stone without additional treatment (15.3%)	P value
**Gender**				0.820
	Male	36 (59.1%)	6 (54.5%)	
	Female	25 (40.9%)	5 (45.5%)	
**Laterality**				0.223
	Right side	27 (44.2%)	4 (36.3%)	
	Left side	34 (55.8%)	7 (63.7%)	
Stone size (mm)		15±4.2	22±4.9	**0.004**
**Stone location**				**0.002**
	Renal pelvis or single calyx	45 (73.7%)	3 (27.2%)	
	Partial/complete staghorn	16 (26.3%)	8 (72.8%)	
**Hydronephrosis Grade**				0.065
	Grade 0-1	22 (36.1%)	5 (45.4)	
	Grade 2-3	39 (63.9%)	6 (54.6)	

## DISCUSSION

The high risk of recurrence of the stones in the pediatric age group and necessity of multiple surgical interventions has led to development of minimally invasive treatment methods with maximal efficiency. ESWL, RIRS, PCNL (micro-, mini-) and laparoscopic surgery are the standard minimal invasive procedures to treat renal stones in pediatric patients.

ESWL is the least invasive and first-line treatment method that is used for the management of pediatric renal stones. Despite its widespread use in adults, ESWL for pediatric renal stones was first performed in 1986 by Newman (9). Reports have showed the safety and efficacy of the ESWL even in low birth weight infants (10). Limited reports about ESWL in infancy exist because of the rarity of renal stones in infancy. The success rate of ESWL in infant patient was 84.6-100% (11, 12). Despite these high stone free rate of the procedure, ESWL has some disadvantages such as requirement of anesthesia, ureteral obstruction in high volume renal stones and higher additional intervention rate (13). In current literature ESWL is a safe method of treatment from the point of view of development of new onset diabetes mellitus and hypertension; however, long term detrimental effect of ESWL on kidney which can cause diabetes mellitus and hypertension is still controversial (12, 14).

With the advancement of small caliber f-URS, management of renal stones in childhood have become possible even in infant patients. Cannon et al. published the first series of the RIRS in pediatric patient (15). Stone free rate in pediatric RIRS studies varies between 76-99%. However age of patient in these series was generally greater than 3 years old (15, 16). Li et al. published first series of RIRS in infant patient with SFR of 94.6%. Ten out of 55 infants underwent simultaneous bilateral RIRS (17). One of the major problems during the procedure in this age group of patient was ureteral access sheath (UAS) insertion. The younger the child was, the harder the insertion of UAS. In reported series of pediatric RIRS, UAS was inserted in 43.7-61.5% of patients (16-18). Li et al. did not use UAS, rather they inserted DJ stent in all patients before the procedure and after a while 8Fr f-URS was advanced over the hydrophilic guidewire (17). Generally, major complications were not observed in these procedures. Urinary system infection, postoperative hematuria, ureteral mucosal injury and ureteral perforation were the most commonly observed complications in pediatric RIRS series (16, 17).

The first mini-PNL series was published in 1998 by Jackman et al. in which they used the 11Fr sheath and 7Fr rigid cystoscope in 11 procedures without any complication (19). Success rate of mini PNL procedure in recent infant series varies between 70.8-92.5% after single session and 81.2-92.5% after additional session. Bodakci et al. showed that stone free rate was 70.8% at the end of the postoperative 24 hour and 81.2% at the end of the first postoperative week in 48 infant mini-PNL procedures (20). Bo Xiao et al. performed all mini-PCNL with ultrasound-guidance in 67 renal units of 56 patients aged <3 years. They found that SFR during the hospital discharge was 92.5% (21). In the retrospective study of Brodie et al. which was conducted in 46 patients under age of 16, 76% of patients achieved 100% stone clearance after a single session of mini-PCNL and 100% of patients achieved stone clearance of greater than or equal to 80% (22). SFR was 76.9% in a prospective study of Kareem Daw et al. and SFR increased to 85% and 92.3% after ESWL and auxiliary therapy respectively (23). Pelit et al. reported a SFR of 84.4% and 91.1% after initial and additional treatment respectively (18). In our study, we reached SFR of 84.7% and 94.4% after single and additional session respectively. Further analysis of SFR revealed that stone size and stone location were factors that affected the stone free status of the patients. In our observation, although hydronephrosis grade did not affect the success of the procedure it facilitated the access to the collecting systems and shortened the operation time. We believe that an SFR of 94.4% for mini PNL procedure in infants is acceptable. In parallel to the literature on mini PCNL in infants, stone free rates of our series after initial and additional therapy is higher than some studies in infant patients. This is because we have an increased experience in childhood urolithiasis due to the location of our hospital in an endemic stone area.

Holmium: yttrium-aluminum-garnet (h-YAG) laser and pneumatic intracorporeal lithotripsy tecniques were both used for stone fragmentation in infant mini-PCNL. As it was in our study, Bodakci et al. used only pneumatic lithotoripsy for fragmentation and SFR was 81.2% in their series (20). Brodie and Bo Xiao et al. used both pneumatic and laser lithotripsy for fragmentation depending on the surgeon's preference and SFR was 76% and 92.5% in their studies, respectively (21, 22). Kareem Daw et al. used only h-YAG laser for stone fragmentation and they obtained a 76.9% of SFR (23). However in current litarature, there was no study that compare the effect of lithotripsy techniques on SFR in infant mini-PCNL.

Although the stone-free rates were higher after mini PNL, complications were not uncommon in infancy, such as bleeding, urosepsis, colon perforation, hypothermia and urinary leakage. Pediatric patients were more likely to bleed during system dilatation because of the fragile renal parenchyma and delicate collecting system. In mini PNL series of infancy, blood transfusion rates were lower than the pediatric age patients and varied between 0% and 7.5% (20, 21). Studies showed that blood transfusion rates were higher with the use of 24-26Fr sheaths than with ≤18Fr (5.9%) sheaths (24). We dilate the renal tract up to 20Fr or 22Fr according to the stone size and age of patients. In our opinion, the 22Fr sheath should be kept for stones larger than 2cm to reduce the bleeding if the patient's age and physical development is appropriate.

Children can easily become hypothermic due to the long-running operations, cold irrigation fluids and operation room (25). Roberts et al. showed that hypothermia is directly related with the duration of the operation and they also stated that preoperative preparation, anesthesia induction and patient positioning contribute the fall of the body temperature as much as the surgical procedure itself (26). In our patients, we did not observe any hypothermic complications. We think that irrigation fluids at body temperature and heater blanket prevent infants from being hypothermic and shortening the duration of all surgical steps including preoperative procedures, anesthesia induction and positioning preserve the core body temperature of the patients.

One of the important issues of mini-PCNL in infants is radiation exposure. Some methods have been tried to minimize radiation exposure. Bo Xiao et al. punctured the collecting system under USG guidance to reduce radiation exposure (21). Bodakci et al. achieved US-guided intrarenal access in 7 of 40 to decrease the fluoroscopy time (22). In all series, lead aprons were used to protect patient's gonads like our studies.

Bowel perforations are also rare complications, however carry high morbidity and mortality risk. It is observed as 0.2% to 0.3%. Dilated collecting tubules, horseshoe kidney, and retro-renal colon are risk factors for colon injury (27). To avoid colon perforation in PCNL, some techniques have been proposed. Ultrasound-guided puncture or CT-guided puncture of the pelvicaliceal system in patients with anatomic abnormalities could prevent colon perforation (28, 29). However, these access techniques were not routinely applied during PCNL procedures. For this reason, especially the left renal lower pole access with other retro-renal colon risk factors, the bowel injury should be considered during PNL. Conservative treatment with drainage of urinary system and gastrointestinal system separately is generally the first choice of method (30). In our patient, we observed colonic content in nephrostomy tube at postoperative 3 day and we decided to repair the colon primarily with pediatric surgeons. The patient was discharged uneventfully at postoperative 9 day following laparotomy.

The overall incidence of hydrothorax after PCNL procedures was between 0% and 3.3%. The risk of injury increased with the supracostal access due to the position of the pleura (31). As in our case that we have performed supracostal puncture, respiratory distress developed on postoperative day 1 and hydrothorax was diagnosed. This patient was managed successfully by intercostal chest tube drainage.

Our study is limited by its retrospective nature. On the other hand, it is strengthened by the high number of patients.

## CONCLUSIONS

Mini-PNL with pneumatic intracorporeal lithotripsy can be performed safely and effectively to manage kidney stones with high stone free rate and low complications in patients under the age of 3. Exposure of infant patients to hypothermia and radiation must be kept in mind during the operation. This method of treatment provides an acceptable SFR in experienced center.
